# The impact of declining vaccination coverage on measles control: a case study of Abia state Nigeria

**DOI:** 10.11604/pamj.2013.15.105.2515

**Published:** 2013-07-22

**Authors:** Chukwuemeka Anthony Umeh, Hycienth Peterson Ahaneku

**Affiliations:** 1 Hospitals Management Board, Bayelsa state Nigeria; 2 Department of Epidemiology, University of Texas School of Public Health, Houston, Texas, USA

**Keywords:** Measles, Immunization, Nigeria

## Abstract

**Introduction:**

Efforts at immunizing children against measles was intensified in Nigeria with nation-wide measles vaccination campaigns in 2005 - 2006, 2008 and 2011 targeting children between 9 and 59 months. However, there were measles outbreaks in 2010 and 2011in Abia state Nigeria. This study seeks to find out if there is any association between measles immunization coverage and measles outbreak.

**Methods:**

This is a descriptive analysis of the 2007 to 2011 Abia state measles case-based surveillance data supplied to Abia state World Health Organization office and Abia State Ministry of Health by the disease surveillance and notification officers.

**Results:**

As the proportion of cases with febrile rash who were immunized decreased from 81% in 2007 to 42% in 2011, the laboratory confirmed cases of measles increased from two in 2007 to 53 in 2011.Of the laboratory confirmed cases of measles, five (7%) occurred in children < 9 months, 48 (64%) occurred in children 9 - 59 months and 22 (29%) occurred in children < 59 months old. Seventy five percent of all laboratory confirmed cases of measles occurred in rural areas.

**Conclusion:**

Efforts should be made to increase measles immunization in children between 9 and 59 months as most cases of measles occurred in this age group as immunization coverage dropped. In addition, further studies should be carried out to determine the cause of the disproportional incidence of measles in rural areas in Abia state bearing in mind that measles immunization coverage in urban and rural areas was not markedly different

## Introduction

Measles is a highly infectious disease that is transferred from one person to another through aerosolized droplets or by direct contact with the nasal and throat secretions of infected persons [1]. Measles transmission is prevented by vaccination and in sub-Saharan Africa, it is recommended that the vaccine be given at 9 months of age, by which time the child would have lost passive immunity conferred by maternal antibodies. One dose of measles vaccine confers life-long immunity to approximately 85% of those vaccinated [1]. Childhood immunization programs targeted at children less than 59 months have led to a marked decrease in measles infections and outbreaks [2]. However, in order to interrupt the endemic transmission of measles virus; a population immunity of ≥95% has to be achieved [2].

Measles case fatality is estimated to be between 3 to 5% in developing countries and may be as high as 10% during epidemics [1]. Despite the efforts made at increasing immunization, measles remain a leading cause of under-five mortality in Africa [3].There were 139, 300 measles deaths globally in 2010 which represents nearly 380 deaths every day or 15 deaths every hour [4]. Nigeria is one of the 45 countries that together account for 94 percent of the global deaths caused by measles [5].

Measles-case based surveillance is a system put in place to detect cases and outbreaks of measles. It involves reporting and investigating any suspected case of measles and to use the data to evaluate immunization efforts and predict outbreaks through the identification of geographical areas and age groups at risk [1]. In 2006, measles case based surveillance was established in Nigeria using the resources and infrastructure of the already established surveillance for Acute Flaccid Paralysis (AFP). It involves both passive and active surveillance [2, 3].

In 2008, the WHO African regionaloffice set aregional pre-elimination goal to be achieved by the end of 2012. The goals include (1) reducing the incidence of measles to < 5 cases/ 106 population per year in all countries, (2) increasing the first dose ofmeasles containing vaccine (MCV1) to greater than 90% at the national level and greater than 80% in all districts and (3) measles surveillance system performance that reports non-measles febrile rash illness rate of ≥2 cases per 100,000 population per year [6].

### Abia State information

Abia state is in the south-eastern part of Nigeria and covers a land area of 5,243.7 square kilometers. The population of Abia State by the 2006 population census was 2,833,999 and with an annual growth rate of 2.7%, the estimated population in 2011 is 3,278,699. It has 17 local governments [7, 8]. About 70% of the population lives in rural areas. The state has a high burden of a young population with children aged 0-14 years accounting for 36.8% of the population. There is also a high age dependency ratio of 66.5%. Over 59% of the population is estimated to live below the poverty line of one US dollar a day [8]. This study analyzes measles case surveillance in Abia state between 2007 and 2011 and looks at the impact of declining routine immunization coverage on measles control

## Methods

Measles cases reported to Abia state World Health Organization (WHO) office and Abia state Ministry of Health from 2007 to 2011 through the Integrated Disease Surveillance and Response were analyzed. The data was collected from 130 disease surveillance focal sites in Abia state. These sites are evenly distributed around the state and are involved in both active and passive surveillance of measles in communities and health facilities. The focal persons at these sites report cases of measles to the local government disease surveillance and notification officers (DSNO). The local government areas DSNOs are expected to collect blood samples from the cases within 30 days of the onset of rash. The local government DSNOs also report clinical and epidemiological data of the measles cases weekly to the state DSNO and the state WHO office. The state epidemiologist forwards the data weekly to the national epidemiology center.

A suspected measles case is defined as any person with fever and generalized maculo-papular skin rash plus cough or conjunctivitis or runny nose. Measles IgM antibodies are used for laboratory confirmation of measles. Since both measles vaccination and measles infection cause increased IgM antibodies, any case with a positive measles IgM who had received measles vaccination 30 days prior to the collection of serum sample is not considered to be a laboratory confirmed case of measles but as a case ofIgM positivity secondary to measles immunization [1, 2].

A confirmed outbreak of measles is defined as 3 or more measles IgM positive (laboratory confirmed) cases in a health facility or district in one month [9]. Measles incidence per 100,000 population was calculated by dividing the number of reported measles cases by the population (based on the 2006 census) and multiplied by 100,000. Data was analyzed using SAS 9.1.

## Results

From Table 1, a total of 757 cases of febrile rash were reported between 2007 and 2011 in Abia state. There were 75 laboratory confirmed cases with 2 cases in 2007, 1 in 2008, none in 2009, 19 in 2010 and 53 in 2011. Measles surveillance showed an improvement between 2007 and 2011. The annual reporting rate of febrile rash illness per 100,000 of population increased from 3.37 in 2007 to 7.23 in 2011(target ≥2). Local governments (districts) that investigated at least 1 case with a blood specimen remained at 100% between 2007 and 2011 (target < 80%).

**Table 1:** Demographics of cases with rash

Variable	2007	2008	2009	2010	2011	2007 - 2011
Reported cases of rash	99	100	176	174	228	757
Reported rash/100,000 population	3.37	3.31	5.73	5.52	7.23	
Measles IgM +ve	2/99 (2.02%)	1/100 (1%)	0/176 (0%)	19/174 (10.92%)	53/228 (23.25%)	75/757 (9.91%)
Measles IgM +ve/1,000,000 population	0.68	0.33	0	6.02	16.81	
**Sex**						
Male	55/99 (55.56%)	60/100 (60%)	74/176 (47.44%)	84/174 (48.28%)	111/228 (48.68%)	
Female	44/99 (44.44%)	40/100 (40%)	82/156 (52.56%)	90/174 (51.72%)	117/228 (51.32%)	
**Age**						
< 9 months	8/99 (8.08%)	15/100 (15%)	10/156 (6.41%)	16/174 (10.76%)	10/228 (4.39%)	59/757 (7.79%)
9 – 59 months	49/99 (49.49%)	50/100 (50%)	78/156 (50%)	86/174 (46.20%)	115/228 (50.44%)	378/757 (49.93%)
> 59 months	42/99 (42.42%)	35/100 (35%)	68/156 (43.49%)	72/174 (43.04%)	103/228 (45.18%)	320/757 (42.27%)
**Residence**						
Rural	75/99 (75.76%)	80/100 (80%)	112/156 (71.79%)	135/174 (77.59%)	189/228 (82.89%)	591/757 (78.07%)
Urban	24/99 (24.24%)	20/100 (20%)	44/156 (28.21%)	39/174 (22.41%)	39/228 (17.11%)	166/757 (21.93%)
Immunized	81/99 (81.82%)	70/100 (70%)	115/156 (73.72%)	98/174 (56.32%)	96/228 (42.11%)	460/757 (60.77%)
9 – 59 months immunized	40/49 (81.63%)	41/50 (82%)	60/78 (76.92%)	55/86 (63.95%)	60/115 (52.17%)	256/378 (67.72%)
Unimmunized by place of residence						
Urban	6/24 (25%)	7/20 (35%)	9/44 (20.45%)	16/39 (41.03%)	19/39 (48.72%)	57/166 (34.34%)
Rural	12/75 (16%)	23/80 (28.75%)	32/112 (28.57%)	60/135 (44.44%)	113/189 (59.79%)	240/591 (40.61%)
Number of confirmed measles outbreak	0	0	0	1	5	6

The analysis of the age of the cases with febrile rash showed that on average over the five years; approximately 50 percent of the cases with febrile rash were within 9 and 59 months. 8% were less than 9 months and the remaining 42% were above 59 months. 64% of confirmed cases of measles between 2007 and 2011 were between 9 and 59 months. 7% were below 9 months and 29% were above 59 months (Table 1).

However, of the confirmed cases of measles between 2007 and 2011, 5 (7%) occurred in children < 9 months, 48 (64%) occurred in children 9 ≥59 months and 22 (29%) occurred in children > 59 months old (Table 2).

**Table 2:** Laboratory confirmed measles cases

	2007 n/N	2008 n/N	2009 n/N	2010 n/N	2011 n/N	2007 – 2011 n/N
**Sex**						
Male		1/1		8/19 (42.11%)	34/53 (64.15%)	384/757 (50.73%)
Female	2/2			11/19 (57.89%)	19/53 (35.85%)	373/757 (49.27%)
**Residence**						
Rural	1/2			13/19 (68.42%)	42/53 (79.25%)	56/75 (74.67%)
Urban	1/2	1/1		6/19 (31.58%)	11/53 (20.75%)	19/75 (25.33%)
**Immunization**						
No	1/2			15/19 (78.95%)	30/53 (56.60%)	46/75 (61.33%)
Yes	1/2	1/1		4/19 (21.05%)	23/53 (43.40%)	29/75 (38.67%)
**Age group**						
<9 months	1/1			2/19 (10.53%)	2/53 (3.77%)	5/75 (6.67%)
9 months – 59 months	1/1	1/1		14/19 (73.68%)	32/53 (60.38%)	48/75 (64%)
>59 months				3/19 (15.79%)	19/53 (35.85%)	22/75 (29.33%)
**Season of the year**						
Dry season	1/2	1/1		13/19	46/53	61/75 (81.3%)
Rainy season	1/2	0		6/19	7/53	14/75 (18.7%)

About 80% of cases with febrile rash reside in the rural areas while about 75% of those with laboratory confirmed cases of measles reside in the rural area (Table 1 and Table 2).

As the measles vaccination in 9 - 59 months old dropped from 81.63% in 2007 to 52.17% in 2011, the confirmed cases of measles/1,000,000 population increased from 0.68 to 16.81. Furthermore, the number of measles outbreak increased from 0 in 2007 to 5 in 2010 as the proportion of the population immunized dropped (Table 1).

On average, between 2007 and 2011, 34% of cases with febrile rash living in urban areas were unimmunized while 41% of those living in rural areas were unimmunized. In 2007, 25% of febrile rash cases living in urban areas and 16% of those living in rural areas were unimmunized while in 2011, 49% of those living in urban areas and 60% of those living in rural areas were not immunized (Table 1).

Over the five years under review, for cases with confirmed cases of measles, 61.33% of them were unimmunized while 87% of the confirmed cases of measles occurred during the dry season (Table 2). The peak of measles infection occurred in January and February which accounted for 55% of all cases (Table 3).

**Table 3:** Showing the distribution of laboratory confirmed cases of measles between 2007 and 2011

Month	Frequency	Percentage
January	21	28%
February	20	26.7%
March	9	12%
April	3	4%
May	1	1.3%
June	5	6.7%
July	2	2.7%
August	1	1.3%
September	1	1.3%
October	1	1.3%
November	8	10.7%
December	3	4%
**Total**	75	100%

## Discussion

As the measles vaccination in 9 - 59 months old dropped from 81.63% in 2007 to 52.17% in 2011, the confirmed cases of measles/1,000,000 population increased from 0.68 to 16.81. Furthermore, the number of measles outbreak increased from zero in 2007 to five in 2010 as the proportion of the population immunized dropped. This resurgence of measles may be due to the decline in immunization coverage in the state or due to other environmental factors that are known to increase communicable diseases such as poor water supply, poor housing and sanitary conditions, food scarcity and climate change [10]. However, there was no major environmental change in the state between 2009 and 2011, making the decline in vaccine coverage the most plausible reason for the measles outbreak.

The result showed that 39.23% of those who had febrile rash between 2007 and 2011 were not immunized. This is low compared to the national average of 59% [9]. 61.33% of the laboratory confirmed cases of measles were not immunized compared to the Nigerian average of 71.2% [9].

Similarly, the proportion of those with febrile rash immunized decreased from 81.82% in 2007 to 42.11% in 2011. Conversely, WHO reported an increase in measles containing vaccine (MCV) vaccination in Nigeria from 41% in 2007 to 71% in 2011 [11]. This decrease in measles vaccination in Abia state might be due to several factors such as vaccine stock out in the state, insecurity and poor funding of vaccination outreaches by the local governments.Failure to maintain high coverage of childhood immunization in all districts has been noted to lead to the resurgence of measles [6, 12].

About 75% of the measles cases occurred in rural areas. Although the average unimmunized rate for the 5 years for urban areas (34%) is not very different from those in the rural areas (41%), there was a wide discrepancy in the rate of cases in urban and rural areas. This discrepancy might be explained by the fact that more people live in rural areas than urban areas. However, another reason might be that children in rural areas are poorer, more malnourished and more susceptible to infection [5]. This means that more efforts in immunization should be concentrated in rural areas.

Most of the cases of measles occurred in the dry season, with the peak in January and February. This is in conformity with earlier observed trend of measles infection [12]. However, in a study in Niger, the cases of measles started in September, increased progressively and peaked in March [13].

## Conclusion

Decline in routine childhood measles immunization could have led to the resurgence of measles in Abia state after the state was measles-free for more than 12 months. Furthermore, most cases of measles occur in children between 9 and 59 months and most cases of measles occur in rural areas. Efforts should be made to increase measles immunization in children between 9 and 59 months. Due to the peak incidence of measles in the dry season vaccination campaigns should be increased towards the end of the rainy season to prevent outbreaks in the beginning of the dry season. Similarly, in countries with limited resources for regular vaccination outreaches to rural communities, measles vaccination outreaches should be planned towards the end of the rainy season in order to capture most of the children who are susceptible to measles before the onset of the dry season when they are more prone to measles infection. Further studies should be carried out to determine the cause of the disproportionate incidence of measles in rural areas in Abia state bearing in mind that measles immunization coverage in urban and rural areas are not markedly different.
